# Supplementary material for: Characterizing A21: Natural Cyanobacteria-Based Consortium with Potential for Steroid Bioremediation in Wastewater Treatment
Source: Int J Mol Sci. 2024 Dec 4;25(23):13018. doi: 10.3390/ijms252313018 (PMC11641370; doi:10.3390/ijms252313018)
Supplement: Supplementary file 1 [file ijms-25-13018-s001.zip › ijms-3358971-supplementary.pdf]

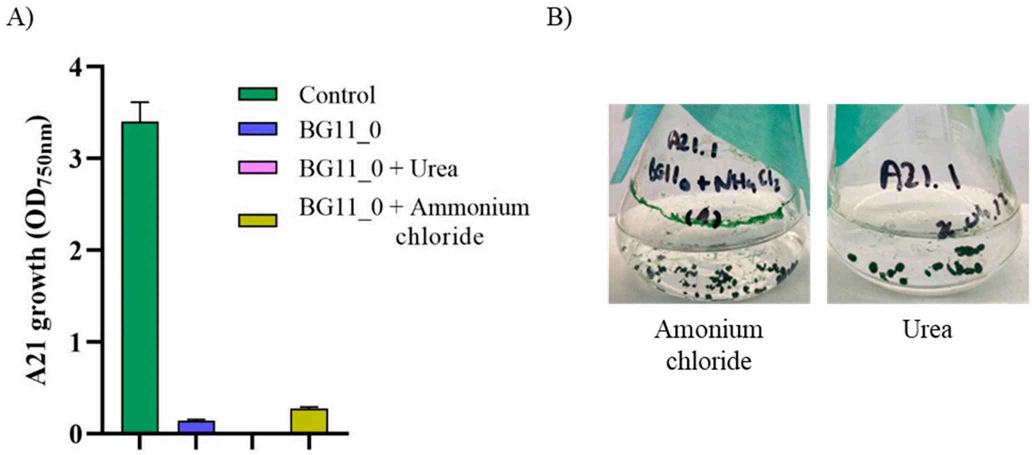

Figure S1. Effect of nitrogen source on A21 growth. NaNO3 in BG11 was substituted for urea or NH4Cl (16 mM). Alternatively, no nitrogen was added (BG110). **A)** Growth graph of A21 after 10 days. The average of the three replicates and their error bars are represented; **B)** Photograph showing the result of growth after 10 days in BG11 with NH_4Cl_2 and the growth form of the strain when it is transferred from the plate to liquid BG11 medium. In all cases, the graphs show the average OD_{750nm} of three biological replicates together with the standard deviation (n=3)

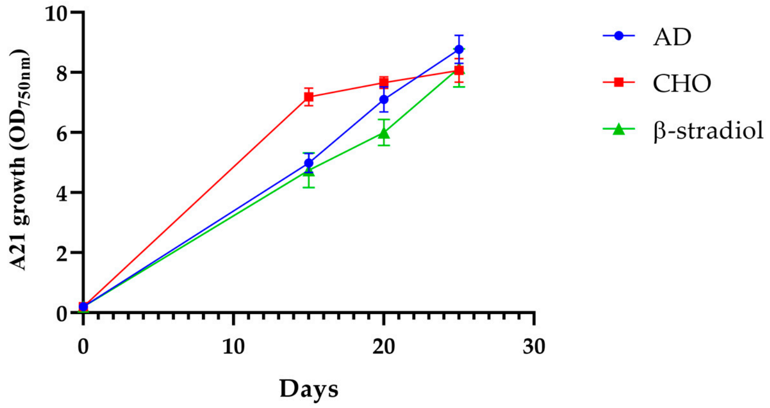

Figure S2. Growth of the A21 consortium in the presence of different steroids. Growth curve of the A21 consortium in BG11 medium supplemented with 4-androsten-3,17-dione (AD), cholesterol (CHO) or β-estradiol (E2) at 0.1 mM over 25 days. The growth was measured at 750nm. Data points represent the mean ± standard deviation from three independent replicates.

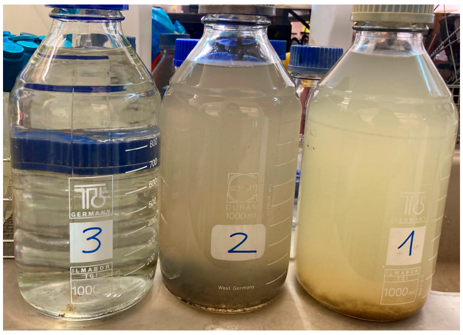

Figure S3. Primary, secondary, and tertiary treatment wastewater from a treatment plant in the Community of Madrid.

Table S1. PCR primers used in this work

Primer	Sequence (5´-3´)	PCR	Reference
	**Cyanobacteria identification, 16S rRNA amplification**		
CYA106-Fw	CGGACGGG GAGTAACGCGTGA	30 cycles (Tm 55ºC, 30 s, 72ºC, 30 s)	[73]
CYA359-Fw	GGGGAATYTTCCGCAATGGG		[73]
CYA781-Rv(a)	GACTACTGGGGTATCTAATCCCAT		[73]
CYA781-Rv(b)	GACTACAGGGGTATCTAATCCCTTT		[73]
	**Identification of metagenomic amplification 16S rRNA**		
16S_V1-V2_illum_Fw	CTACACGACGCTCTTCCGATCTTCTTTGGCTCAGGACGAACGCTGGCGGC	30 cycles (Tm 55ºC, 30 s, 72ºC, 30 s)	[65]
16S_V1-V2_illum_Rv	GTGACTGGAGTTCAGACGTGTGCTCTTCCGATCTCTTCCTACTGCTGCCTCCCGTAGGAGT		[65]
16S_V3-V4_illum_Fw	CTACACGACGCTCTTCCGATCTTCTTCCTACGGGNGGCWGCAG		[64]
16S_V3-V4_illum_Rv	GTGACTGGAGTTCAGACGTGTGCTCTTCCGATCTCTTGACTACHVGGGTATCTAATCC		[64]
P5_index	AATGATACGGCGACCACCGAGATCTACACXXXXXXXXACACTCTTTCCCTACACGACGCTC	15 cycles (Tm 55ºC, 30 s, 72ºC, 30 s)	Nextera (Illumina)
P7_index	CAAGCAGAAGACGGCATACGAGATCGTGATXXXXXXXXGTGACTGGAGTTCAGACGTGT		Nextera (Illumina)
	**Identification of eukaryotic microorganisms, 18S rRNA amplification**		
ss5-Fw	GGTGATCCTGCCAGTAGTCATATGCTTG	Tm 55°C, 30 s, 72°C, 120 s,	[74]
ss3-Rv	GATCCTTCCGCAGGTTCACCTACGGAAACC		
18S-Fw	GTCAGAGGTGAAATTCTTGGATTTA	Tm 56°C, 30 s, 72°C, 120 s,	[75]
18S-Rv	AGGGCAGGGACGTAATCAACG		

Reverse primer CYA781R was an equimolar mixture of CYA781R(a) and CYA781R(b).

X: P5_S502 CTCTCTAT; P5_S503 TATCCTCT; P5_S505 GTAAGGAG; P5_S506 ACTGCATA; P5_S507 AAGGAGTA; P5_S508 CTAAGCCT; P5_S510 CGTCTAAT; P5_S511 TCTCTCCG; P7_N710 CGAGGCTG; P7_N711 AAGAGGCA; P7_N712 GTAGAGGA; P7_N714 GCTCATGA; P7_N715 ATCTCAGG; P7_N716 ACTCGCTA; P7_N718 GGAGCTAC; P7_N719 GCGTAGTA

Table S2. Statistical analysis (see Excel).

Table S3. Carbon source Utilization by A21 under dark conditions. The table summarizes the growth of strain A21 in BG11 medium supplemented with various carbon sources, assessed under dark conditions. The BG11 control was incubated under light as a reference.

**Carbon Source**		**A21.1**
Control BG11		+
**Monosaccharides**	Glucose	-
	Fructose	-
	Mannose	-
	Galactose	-
	Arabinose	-
**Disaccharides**	Sucrose	-
	Maltose	-
	Lactose	-
**Alcohol**	Glycerol	-

Growth is indicated as positive (+) or negative (-).

Table S4. Antibiotic sensitivity of A21 consortium

	**Km**				**Cm**				**Spt**				**Spm**			
**Isolated**	20	50	75	100	7.5	15	30	75	2	4	8	20	2	4	8	20
**A21**	-	-	-	-	-	-	-	-	-	-	-	-	-	-	-	-

	**Em**				**Gm**				**Nal**				**Nm**			
**Isolated**	20	40	80	200	2	4	8	20	7.5	15	30	75	25	50	100	250
**A1**	-	-	-	-	+	+	+	-	-	-	-	-	-	-	-	-

The concentrations shown for each antibiotic correspond to µg/mL. +: robust growth; +/-: some growth; -: no growth. Antibiotics: kanamycin (Km), chloramphenicol (Cm), spectinomycin (Spt), streptomycin (Spm), erythromycin (Em), gentamicin (Gm), nalidixic acid (Nal), and neomycin (Nm).

Table S5. Plasmids used for the triparental mating.

**Plasmid**	**Description**	**Reference**
pRK2013	Conjugative plasmid, derived from RK2. Ori: *ColE1*. Km^R	Addgene
pRL623	Helper plasmid. It contains the M.*AvaI*, M.*Eco47II*, M.*EcoT22I* methylases and mob_{ColK} gene. Cm^R	[76]
pSEVA221	Cargo plasmid. Ori: RK2. Km^R	[77]
pSEVA231	Cargo plasmid. Ori pBBR1. Km^R	
pSEVA241	Cargo plasmid. Ori pRO1600/ColE1. Km^R	
pSEVA251	Cargo plasmid. Ori RSF1010. Km^R	
pSEVA251-Cpf1	Cargo plasmid. Ori RSF1010. It contains CRISPR-Cpf1 nuclease. Km^R	[57]
pSL2680	Cargo plasmid. Ori RSF1010. It contains CRISPR-Cpf1 nuclease. Km^R	[78]
